# Triglyceride profiles in preterm infants reveal dynamic and age-specific trajectories

**DOI:** 10.3389/fped.2026.1777798

**Published:** 2026-06-12

**Authors:** Emma R. Gertel, Victoria Y. Ding, Thanaphong Phongpreecha, Feng Xie, Andrew Parsons, Ivana Marić, Nima Aghaeepour, Karl G. Sylvester, Valerie Y. Chock, John A. Kerner, Camilia R. Martin, David K. Stevenson, Gary M. Shaw, Jonathan D. Reiss

**Affiliations:** 1 Sackler School of Medicine, American Medical Program, Tel Aviv University, Tel Aviv, Israel; 2 Stanford Quantitative Sciences Unit, Palo Alto, CA, United States; 3 Department of Anesthesiology, Pain and Perioperative Medicine, Stanford University, Stanford, CA, United States; 4 Division of Computational Health Sciences, Department of Surgery, University of Minnesota, Minneapolis, MN, United States; 5 Department of Pediatrics, Division of Neonatology, Stanford University, Stanford, CA, United States; 6 Stanford Department of Surgery, Division of Pediatric Surgery, Stanford University, Palo Alto, CA, United States; 7 Stanford Department of Pediatrics, Division of Gastroenterology, Stanford University, Palo Alto, CA, United States; 8 Department of Pediatrics, Division of Neonatology, Weill Cornell Medicine, New York, NY, United States

**Keywords:** birth weight, gestational age, lipids, NICU, preterm infants, triglycerides

## Abstract

**Introduction:**

Triglyceride (TG) values are routinely monitored in preterm infants receiving lipid emulsion therapy, but their clinical utility remains poorly defined. The objective of this study was to define TG values and their associations with gestational age (GA), birth weight (BW), lipid dosing and postnatal age in preterm infants born <32 weeks' across two distinct academic medical centers.

**Methods:**

We conducted a retrospective multi-centered observational study using data from the Lucile Packard Children's Hospital (LPCH) Stanford Neonatal Intensive Care Unit and the Beth Israel Deaconess Medical Center (BIDMC) Neonatal Intensive Care Unit. TG values in infants <32 weeks' GA were interrogated by GA, postnatal age, BW and in aggregate. Pre- and postnatal factors were evaluated using multivariable logistic regression in the LPCH Stanford data set for associations with hypertriglyceridemia, defined using thresholds of 200 mg/dL and 250 mg/dL. Spline regression was used to model the relationship between continuous TG values and postnatal outcomes in both data sets.

**Results:**

TG distributions differed significantly by GA, with the highest values observed among infants born at 22–25 weeks' gestation and/or with extremely low birth weight. In the LPCH 22–25 week cohort, TG levels peaked at approximately two weeks of postnatal age and subsequently declined. Lipid emulsion dosing <2 g/kg/day was associated with a reduced risk of hypertriglyceridemia, whereas dosing >2 g/kg/day did not significantly alter risk. Continuous TG values were not consistently associated with major neonatal outcomes, including respiratory distress syndrome (RDS), bronchopulmonary dysplasia (BPD), necrotizing enterocolitis (NEC), or retinopathy of prematurity (ROP) in either cohort.

**Discussion:**

TG values in preterm infants vary by developmental stage, closely reflecting GA, BW, and postnatal age, suggesting that circulating TG levels are developmentally regulated. These findings support consideration of age- or weight-based TG reference ranges, particularly for infants born at 22–25 weeks' gestation. In contrast, the limited association between TG levels and neonatal morbidities raises questions regarding the relevance of early TG measurements for predicting later disease.

## Introduction

Preterm infants require specialized care within neonatal intensive care units (NICUs). In the early postnatal period, infants born <32 weeks' gestational age are unable to receive adequate nutrition through enteral milk alone and require parenteral nutrition, including intravenous lipid emulsions to meet their nutritional requirements. In many NICUs, serum triglyceride (TG) levels are routinely measured in infants receiving intravenous lipid emulsions to assess lipid metabolism and clearance from the circulation. However, appropriate 1) TG reference ranges for infants <32 weeks' gestational age (GA) remain unknown; 2) evidence-based definitions for hypertriglyceridemia are lacking; and 3) associations between TG levels and neonatal outcomes are incompletely characterized.

TG ranges for adults and children are based on the guidelines from the American College of Cardiology (ACC) and the American Heart Association (AHA), as detailed in their 2018 guideline on the management of blood cholesterol (1). These ranges are widely accepted and are accompanied by extensive recommendations by the AHA, ACC, and European Society of Cardiology (ESC) on how to best manage TG levels for cardiovascular disease prevention (2). In pediatrics, a combination of expert and society recommendations guide TG reference ranges and definitions for triglyceride reference ranges and associated hypertriglyceridemia. Kerner and Poole recommend TG levels of 150–200 mg/dL for neonates, independent of gestational age at delivery (3). The European Society for Paediatric Gastroenterology, Hepatology, and Nutrition (ESPGHAN) has recommended TG reference ranges remain below 265 mg/dl for all infants (4). For levels >265 mg/dl, it is recommended that intravenous lipid emulsions are temporarily withheld, while maintaining vigilance against essential fatty acid deficiency (5). The American Society for Parenteral and Enteral Nutrition (ASPEN) defines hypertriglyceridemia as >200 mg/dl for any patients, again independent of GA or other factors, receiving parenteral nutrition (6). Despite these recommendations, there are no widely established reference ranges for TG levels in preterm infants <32 weeks GA despite routine administration of intravenous lipid emulsions (7).

Preterm infants <32 weeks of gestation have unique physiologic and metabolic demands that predispose them to TG metabolism that is distinct from older neonates (8, 9). Lipoprotein lipase, the principle enzyme responsible for intravenous TG metabolism, has reduced function and activity in preterm infants (7, 10). Limited adipose stores impair sequestering and mobilization of TG and corresponding fatty acids (7, 8). During periods of stress, beta-oxidation of fatty acids is underdeveloped, limiting energy generation from extracellular lipids (7). Moreover, superimposed clinical events (e.g., sepsis, respiratory distress syndrome, growth restriction) unique to preterm infants may exacerbate such biologic challenges.

The objective of this study was to comprehensively define TG values in preterm infants <32 weeks' gestation, identify early factors associated with hypertriglyceridemia and explore infant outcomes as a function of TG levels. We hypothesized that TG values would inversely correlate with GA, BW and would associate with various neonatal and/or infant outcomes at predefined thresholds of >200 mg/dL or > 250 mg/dL. Here, we report data across two NICUs including the Lucile Packard Children's Hospital (LPCH) Stanford and at the Beth Israel Deaconess Medical Center (BIDMC) Neonatal Intensive Care Unit via the publicly accessible Medical Information Mart for Intensive Care (MIMIC) IV v1.0 database (11, 12).

## Methods

### Lucile packard children's hospital Stanford cohort

This retrospective observational study evaluated infants born <32 weeks' gestation admitted to the NICU at LPCH Stanford between April 2014 and December 2021. All data were extracted and interrogated under an active LPCH Stanford approved IRB protocol with a waiver of informed consent. Ethical approval was obtained as part of the IRB approval process. LPCH Stanford is a level IV NICU with a 70-bed capacity, approximately 5,000 deliveries/year and 1,000 admissions/year. Infants with surgical needs (i.e., necrotizing enterocolitis) are operated upon at LPCH Stanford. Clinical and laboratory data were obtained from the Stanford Research Repository (STARR), which integrates electronic health record information from Stanford's Epic system. TG values and lipid emulsion dosing were linked with individual patients by postnatal day. Clinical diagnoses of severe intraventricular hemorrhage (IVH), necrotizing enterocolitis (NEC), bronchopulmonary dysplasia (BPD), and retinopathy of prematurity (ROP) were primarily identified through International Classification of Diseases (ICD) coding and confirmed by manual chart review for accuracy. Respiratory distress syndrome was defined based on the administration (yes/no) of exogenous surfactant replacement therapy following endotracheal tube placement.

### LPCH Stanford feeding, ILE dosing protocols and TG measurements

All infant nutrition and feeding practices were guided by extremely low birth weight and very low birth weight protocols for the first 7–10 days of life (Supplementary Tables S1, S2). During the study period, all infants <1,500 g received either 20% Intralipid® or SMOFlipid®. Extremely low birth weight neonates received intravenous lipid emulsion (Intralipid® 20% or SMOFlipid) at 0.5 g/kg/day with subsequent increases in 0.5 g/kg/day increments until a target dose of 3.0 g/kg/day was reached. For very low birth weight infants, intravenous lipid emulsions were begun at 1 g/kg/day and advanced in 0.5 g/kg/day increments to a goal of 3 g/kg/day. TG values were routinely obtained as part of daily total parenteral nutrition labs on postnatal days 1–5 and weekly thereafter while on parenteral nutrition.

### Beth Israel deaconess medical center cohort

The Medical Information Mart for Intensive Care (MIMIC) IV v1.0 data set was used to query preterm infants admitted to the Beth Israel Deaconess Medical Center (BIDMC) Neonatal Intensive Care Unit between 2008 and 2019 (11–13). All data were extracted and interrogated under an active BIDMC approved IRB protocol with a waiver of informed consent. Ethical approval was obtained as part of the IRB approval process. BIDMC is a level III NICU with a 60-bed capacity, approximately 5,000 deliveries/year and 1,000 admissions/year. Infants with surgical complications related to prematurity are transferred in the peri-operative period to Boston Children's Hospital and typically returned to the BIDMC NICU following surgery for recovery and convalescence. MIMIC is a freely accessible database of de-identified medical records with accompanying hospital and clinical data. Preterm infants <32 weeks' GA or <1,500 g were identified using ICD-9 and ICD-10 coding (Supplementary Table S3). GA was obtained in 1-week intervals using available ICD codes. BW was available in 250 g intervals through ICD codes. Demographic information, BW, and TG values throughout the initial hospitalization were retrieved and linked. TG values were retrieved with intentional date shifting in place to protect patient privacy. Routine physician ICD coding for all newborns was confirmed by a second individual as part of clinical workflows at the BIDMC NICU. Clinical diagnoses of NEC, BPD, and ROP were primarily identified through ICD coding. Respiratory distress syndrome was also defined based on the administration (yes/no) of exogenous surfactant replacement therapy retrieved from the medication administration record.

### BIDMC feeding and ILE dosing protocols

At BIDMC extremely low birth weight neonates received intravenous lipid emulsion (Intralipid® 20%) at 1.0 g/kg/day on day of life zero and increased to 1.5 g/kg/day on day of life 1. Subsequent increases were made in 0.5 g/kg/day increments until a target dose of 3.0 g/kg/day was reached. For very low birth weight infants, the same target dose and starting protocol were used; however, dose escalations were performed in 1.0 g/kg/day increments. Serum TG concentrations were measured on the day of lipid initiation and subsequently every 1 to 3 days until the target lipid dose was achieved. Thereafter, monitoring was conducted weekly.

Both NICU sites included have similar clinical metrics and practice strategies in terms of the care provided for <32 week infants. This includes factors such as patient census, deliveries, admissions, patient acuity, ventilator management strategies for preterm infants, and feeding practices and protocols.

### Statistical analysis

Neonatal and antenatal characteristics, overall and stratified by complete weeks of 22–24, 25–26, 27–28, 29–30, and >31 weeks), were summarized as counts with percentages or means with standard deviation (SD). The number of TG values per infant, overall and within the first 14 postnatal days, were summarized as medians with interquartile range.

For the LPCH Stanford cohort, serum TG trajectories over the first 14 postnatal days by GA strata were visualized using locally estimated scatter plot smoothing (LOESS), with the degree of smoothing determined by the span value that minimizes the mean squared error via 10-fold cross-validation. Uncertainty around LOESS estimates was visualized using pointwise 95% confidence intervals. Comparisons between GA and BW groups were performed using the Wilcoxon rank-sum test. This analysis was not performed in the BIDMC cohort, due to missing postnatal date availability for each TG value.

For the LPCH Stanford cohort, a multivariable logistic regression model was used to estimate the association of hypertriglyceridemia and maternal and/or infant characteristics through the first 8 postnatal days (informed by lipid emulsion schedule), with hypertriglyceridemia defined using two thresholds (>200 mg/dL, based on ASPEN guidelines, and >250 mg/dL, approximating the ESPGHAN guidelines to not exceed 265 mg/dL). Adjusted odds ratios with 95% confidence intervals were computed. Severe IVH was interrogated as a predictor variable in this analysis, given its known associations with critical illness that can contribute to impaired lipid metabolism (14, 15). Preterm labor was included as a predictor variable given its association wth maternal infectious and/or inflammatory processes, which in turn can alter fetal triglyceride metabolism (7, 8, 16). Preeclampsia was included as a predictor variable due to its associations with relative fetal hypoxia, perturbed amino acid metabolism and concomitant growth restriction (17–20). The multivariable logistic regression was not performed in the BIDMC cohort.

In exploratory analyses, natural spline regression was also used to model nonlinear relationships between TG levels and outcome risk with adjustments for GA (1-week intervals), birth weight (kg) and sex. To align spline regression modeling with continuous predictor variables between both cohorts, continuous BW data for the BIDMC cohort was obtained by interrogating initial caffeine citrate dosing information. A loading dose of 20 m/kg of caffeine citrate is typically and routinely administered to all preterm infants <1,250 g on day of life zero across all NICUs in the United States (21). Maintenance dose data were then used to precisely calculate an infant's birth weight. To address missing GA or BW values, we applied a simple linear regression-based imputation approach. First, a linear regression model was fitted to predict GA from BW using only complete cases with non-missing GA and BW values. This model was then used to impute GA for observations with missing GA values (GA∼BW). Next, a separate linear regression model was fitted to predict BW from GA (BW∼GA), again using complete cases, and used to impute missing BW values. The final imputed variables retained original values when present and substituted imputed values only when missing. For hypothesis testing, a two-sided *p*-value of <0.05 was considered statistically significant. All statistical analyses were conducted using R (version 2025.0.5.0).

## Results

**Figure 1:**
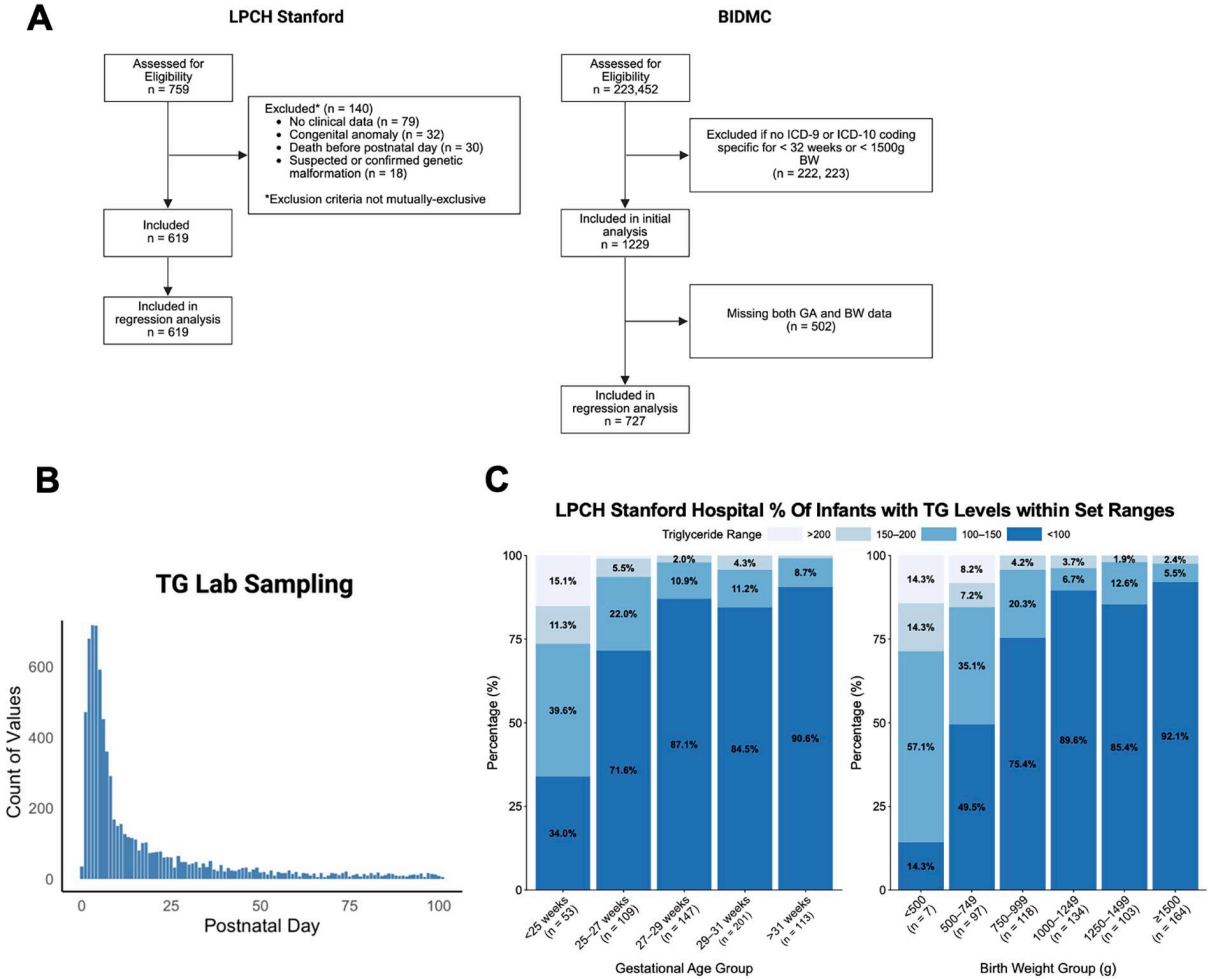
**(A)** STROBE diagram of inclusion criteria for the LPCH Stanford and BIDMC cohorts. **(B)** Histogram of the timing of triglyceride lab sampling by postnatal day (LPCH Stanford cohort only). 69% of triglyceride values obtained occurred within the first 14 days of postnatal life **(C)** Stacked bar plot of % of infants within TG levels (mean, first 14 days) by gestational age and birth weight (LPCH Stanford.

**Figure 2:**
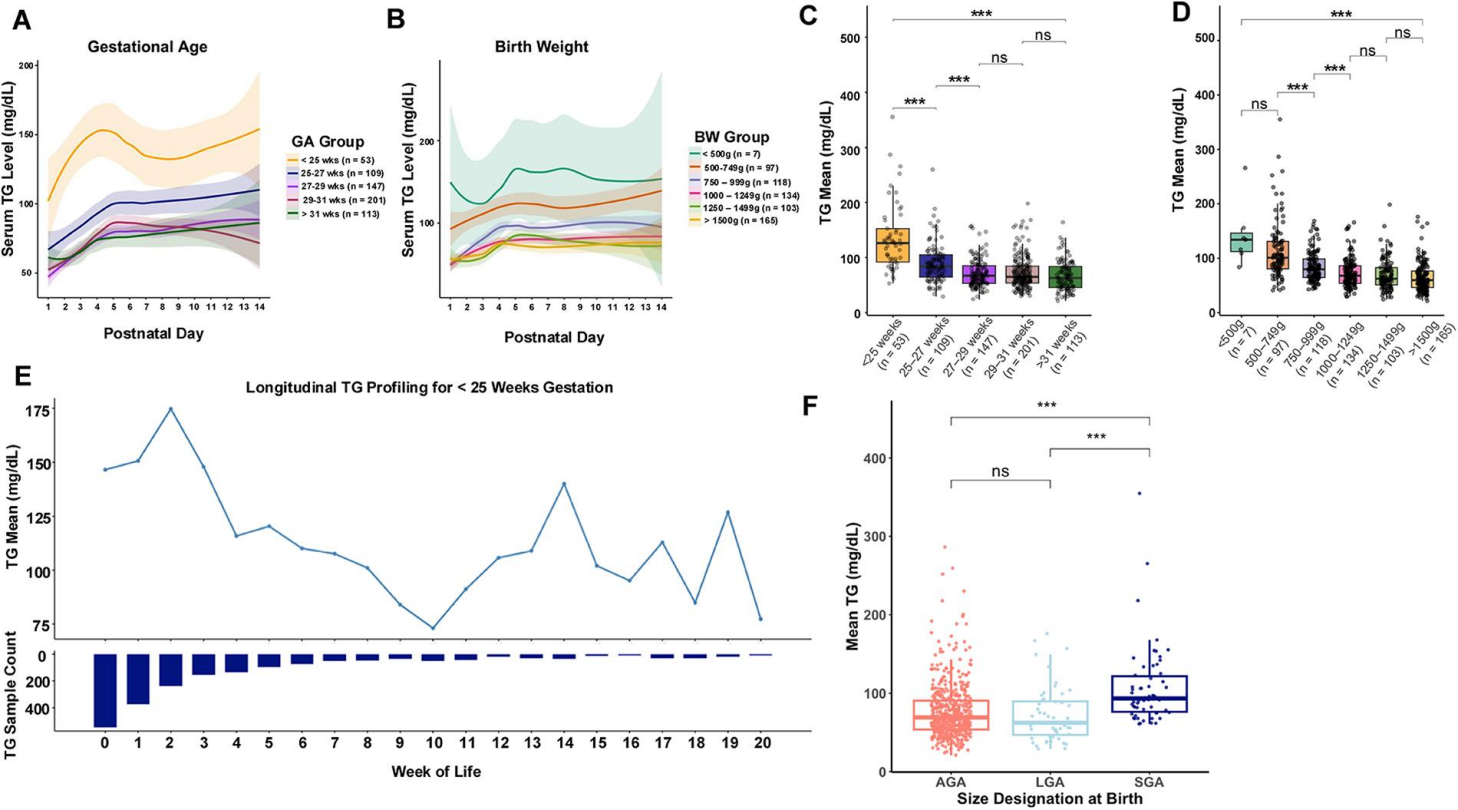
Longitudinal triglyceride profiling in the LPCH Stanford cohort exhibits unique gestational age and birth weight dependent differences. **(A)** Triglyceride profiling (mean) across the first two weeks postnatal age exhibits age-dependent values with the greatest values occurring consistently in the <25 week age cohort. **(B)** Triglyceride profiling across the first two weeks postnatal age exhibits weight-dependent values with the greatest values occurring consistently in the extremely low birth weight category. **(C)** There are significant gestational age and **(D)** birth weight differences across the cohorts. *p-value < 0.05, ** < 0.005, *** < 0.0005, with Wilcoxon-rank sum testing. **(E)** Subcohort analysis of infants <25 weeks’ gestation at birth with longitudinal triglyceride (mean) values vs. week of life. Inverted histogram depicts the relative frequency of the number of triglyceride values obtained in this cohort. TG values rise in the first two weeks of life and then gradually fall until a nadir at 10 weeks of life. **(F)** TG values (mean, first 14 days of life) by size designation at birth (Fenton) suggests that small for gestational age infants (all gestational age strata) have higher TG values compared to their appropriate or large for gestational age counterparts. ns = not significant. *p-value < 0.05, ** < 0.005, *** < 0.0005, with Wilcoxon-rank sum testing.

The initial LPCH Stanford cohort included 759 infants. Infants were excluded for missing clinical data (*n* = 79), a suspected or confirmed congenital anomaly or genetic syndrome (*n* = 50), and/or if death occurred prior to postnatal day 14 (*n* = 30). 619 infants in the Stanford cohort were included in the final analysis (Figure 1A). 69% of TG measurements were obtained within the first 14 days of life (Figure 1B). Most infants (94.7%) received Intralipid® 20% (Table 1). The aggregated LPCH Stanford cohort data had a mean gestational age of 28.5 weeks (SD 2.4 wks). The mean and medium serum TG concentrations were 92.6 mg/dL (SD 69), and 75.0 mg/dL (IQR 64) respectively. 95% of mean values fell between 33.4 mg/dL—165.1 mg/dL. For infants <25 weeks' gestation at birth, 95% of mean values were 63.0 mg/dL—206.7 mg/dL. There were 5,146 discrete TG values obtained in the first 14 postnatal days (Figure 1C), of which 6.6% were >200 mg/dL and 3.4% were >250 mg/dL. There were 99 patients (15.9%) with a TG value >200 mg/dL. A small subset of these patients (18/99) had TG values >200 mg/dL on consecutive days. The average number of days with a TG level >200 mg/dL was 1.73. TG concentrations were visualized by GA and BW using locally LOESS curves, with associated 95% confidence bands (Figures 2A,B). There was a statistically significant inverse relationship between TG values and GA and BW (Figures 2C,D) across the majority of cohorts (<25 weeks vs. 25–27 weeks *p* = 5.93 × 10^−8^, 25–27 weeks vs. 27–29 weeks, *p* = 3.95 × 10^−6^, 27–29 weeks vs. 29–31 weeks, *p* = 9.6 × 10^−1^, 29–31 weeks vs. >31 weeks, *p* = 9.4 × 10^−2^, <25 weeks vs. >31 weeks *p* = 9.40 × 10^−16^. For BW, results demonstrated (<500 g vs. 500 g–749 g, *p* = 0.06, 500 g–749 g vs. 750 g–999 g, *p* = 1.8 × 10^−15^, 750 g–999 g vs. 1,000 g—1,249 g *p* = 3 × 10^−9^, 1,000 g–1,249 vs. 1,250 g–1,499 g *p* = 0.2, 1,250 g–1,499 g vs. > 1,500 g, *p* = 0.15 < 500 g vs. > 1,500 g *p* = 3.2 × 10^−11^. In the sub-cohort of LPCH Stanford neonates born <25 weeks’ gestation, serum TG concentrations were greatest during the first two weeks after birth**.** Levels decreased by 3 weeks postnatal age and reached a nadir at 10 weeks postnatal age (Figure 2E). SGA infants had elevated TG values compared to their AGA and LGA counterparts (Figure 2F).

**Table 1:** LPCH Stanford cohort patient characteristics.

Characteristic	Overall, *N* = 619	Completed Gestational Week at Birth				
		22–24, *N* = 53	25–26, *N* = 108	27–28, *N* = 146	29–30, *N* = 185	31, *N* = 127
Gestational age (weeks), mean (SD)	28.6 (2.4)	24.0 (0.7)	26.0 (0.6)	27.9 (0.6)	30.1 (0.6)	31.5 (0.3)
Birth weight (grams), mean (SD)	1,207 (463)	693 (353)	854 (385)	1,090 (401)	1,387 (357)	1,595 (268)
Sex, *n* (%)						
Female	292 (47.2)	26 (49.1)	56 (51.9)	68 (46.6)	78 (42.2)	64 (50.4)
Male	327 (52.8)	27 (50.9)	52 (48.1)	78 (53.4)	107 (57.8)	63 (49.6)
Race, *n* (%)						
White	133 (24.7)	6 (12.5)	17 (20.0)	35 (28.0)	39 (24.1)	36 (30.3)
Asian	92 (17.1)	6 (12.5)	15 (17.6)	21 (16.8)	31 (19.1)	19 (16.0)
Black	13 (2.4)	2 (4.2)	2 (2.4)	4 (3.2)	2 (1.2)	3 (2.5)
Pacific Islander	10 (1.9)	2 (4.2)	0 (0.0)	3 (2.4)	2 (1.2)	3 (2.5)
Native American	2 (0.4)	0 (0.0)	0 (0.0)	0 (0.0)	1 (0.6)	1 (0.8)
Other	289 (53.6)	32 (66.7)	51 (60.0)	62 (49.6)	87 (53.7)	57 (47.9)
Unknown	80	5	23	21	23	8
Ethnicity, *n* (%)						
Hispanic/Latino	244 (44.7)	26 (54.2)	46 (54.1)	59 (46.1)	70 (42.7)	43 (35.5)
Non-Hispanic	302 (55.3)	22 (45.8)	39 (45.9)	69 (53.9)	94 (57.3)	78 (64.5)
Unknown	73	5	23	18	21	6
Preterm premature rupture of the membranes, *n* (%)	256 (41.4)	34 (64.2)	35 (32.4)	68 (46.6)	75 (40.5)	44 (34.6)
Chorioamnionitis, *n* (%)	111 (17.9)	14 (26.4)	27 (25.0)	29 (19.9)	20 (10.8)	21 (16.5)
Preterm labor, *n* (%)	277 (44.7)	31 (58.5)	61 (56.5)	67 (45.9)	77 (41.6)	41 (32.3)
Preeclampsia, *n* (%)	130 (21.0)	3 (5.7)	21 (19.4)	27 (18.5)	41 (22.2)	38 (29.9)
Fetal growth restriction, *n* (%)	120 (19.4)	4 (7.5)	19 (17.6)	26 (17.8)	47 (25.4)	24 (18.9)
Exposure to prenatal steroids, *n* (%)	606 (97.9)	52 (98.1)	106 (98.1)	144 (98.6)	180 (97.3)	124 (97.6)
Small for gestational age, *n* (%)	50 (8.1)	3 (5.7)	10 (9.3)	10 (6.8)	15 (8.1)	12 (9.4)
Bronchopulmonary dysplasia, *n* (%)	261 (42.2)	45 (84.9)	87 (80.6)	80 (54.8)	40 (21.6)	9 (7.1)
Necrotizing enterocolitis, *n* (%)	44 (7.1)	13 (24.5)	17 (15.7)	7 (4.8)	6 (3.2)	1 (0.8)
Stage 2 retinopathy of prematurity, *n* (%)	86 (13.9)	26 (49.1)	35 (32.4)	18 (12.3)	6 (3.2)	1 (0.8)
Severe intraventricular hemorrhage, *n* (%)	35 (5.7)	12 (22.6)	9 (8.3)	8 (5.5)	4 (2.2)	2 (1.6)
None of the above, *n* (%)	301 (48.6)	4 (7.5)	15 (13.9)	53 (36.3)	124 (67.0)	105 (82.7)
Lipid emulsion type, *n* (%)						
Intralipid® 20%	584 (94.7)	47 (88.7)	98 (90.7)	136 (93.2)	180 (97.3)	123 (98.4)
SMOFlipid® 20%	33 (5.3)	6 (11.3)	10 (9.3)	10 (6.8)	5 (2.7)	2 (1.6)
Unknown	2	0	0	0	0	2

**Figure 3:**
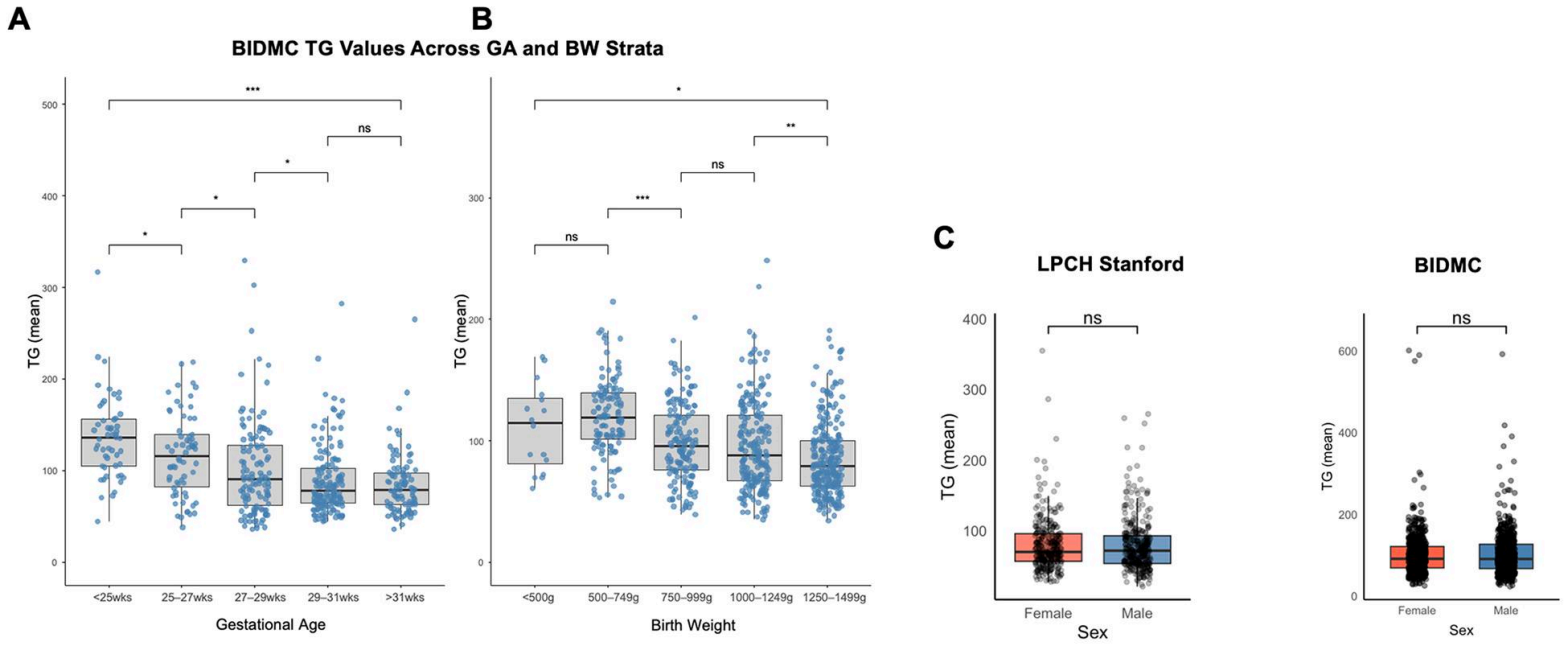
The BIDMC data set (*n* = 1,229) demonstrates similar triglyceride values (mean) with **(A)** gestational and **(B)** birth weight differences across cohorts, **(C)** there was no statistically significant difference in triglyceride values between males and females in either the LPCH Stanford or the BIDMC cohorts. *p-value < 0.05, ** < 0.005, *** < 0.0005, Wilcoxon-rank sum testing.

In the BIDMC cohort, there were 1,229 infants <32 weeks' gestation included in the initial analysis. After pre-processing to determine the number of infants with available continuous GA and BW data, there were 248 with BW data alone, 50 with only GA data alone, and 429 with both GA and BW data available. Thus, 298 infants had either BW or GA data imputed in regression analyses. There were 502 patients with missing GA and BW data that were subsequently excluded. Ultimately there were 727 infants available for the spline regression analysis (Table 2). The mean and median serum TG concentration were 106.30 mg/dL (SD 57.36) and 91 mg/dL (IQR 56), respectively. Among the entire cohort, 95% of mean values fell within 41 mg/dL—197.7 mg/dL. When evaluating infants born <25 weeks' gestation, 95% of mean values were 53.4 mg/dL—217.7 mg/dL. There was a similar inverse relationship between TG values and both GA and BW (Figures 3A,B). This trend was statistically significant across the majority of the GA comparisons (<25 weeks vs. 25–27 weeks, *p* = 0.018, 25–27 weeks vs. 27–29 weeks, *p* = 7.9 × 10^−3^; and 27–29 wks vs. 29–31 wks: *p* = 0.04, 29–31 wks vs. >31 wks, *p* = 0.99 <25 week infants had significantly higher mean TG levels compared to those in the highest GA group (>31 weeks, *p* = 6.9 × 10^−12^). Similarly, mean TG values were significantly higher in the lower BW group (<500 g) compared to larger infants (<500 g vs. 500–749 g, *p* = 0.25, 500–749 g vs. 750–999 g, *p* = 2.1 × 10^−8^, 750–999 g vs. 1,000–1,249 g, *p* = 0.20, 1,000–1,249 g vs. 1,250–1,499 g, *p* = 4.5 × 10^−3^, <500 g vs. 1,250–1,499 g, *p* = 5.9 × 10^−3^). There was no significant difference in either cohort between TG values and infant sex (Figure 3C).

**Table 2:** Characteristics of infants delivered at LPCH Stanford and BIMDC.

Characteristics	Stanford	BIDMC
No. patients	*N* = 619	*N* = 727
Gestational Age (weeks), mean (SD)	28.6 (2.4)	27.6 (2.1)
Birth weight (kg), mean (SD)	1.21 (0.46)	1.05 (0.34)
Triglyceride, mean (SD)	93 (69)	100.6 (52)
Sex (%)		
Female	292 (47.2)	359 (49.4)
Male	327 (52.8)	368 (50.6)
Infant Outcomes		
Respiratory distress syndrome (%)	290 (46.8)	137 (18.8)
Bronchopulmonary dysplasia (%)	261 (42.2)	188 (25.9)
Necrotizing enterocolitis (%)	44 (7.1)	67 (9.2)
Retinopathy of prematurity (%)	86 (13.9)	195 (26.8)

**Figure 4:**
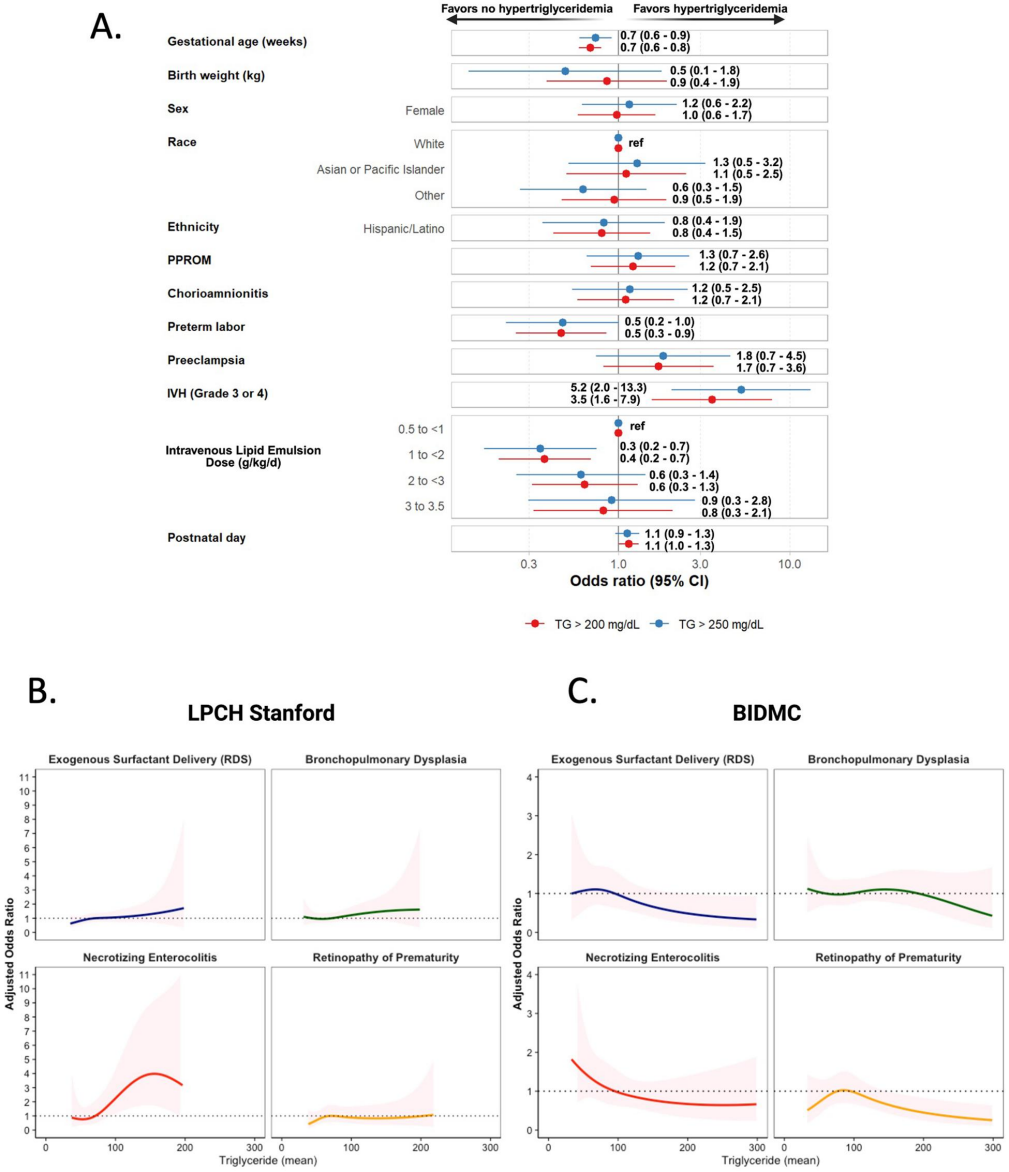
**(A)** logistic regression and associated forest plot in the Stanford/LPCH cohort evaluating predictor variables for associations with hypertriglyceridemia defined as level >200 mg/dL and >250 mg/dL. Lipid emulsion dosing between 1 to <2 mg/kg/d was associated with a lower risk of hypertriglyceridemia compared to higher doses. Higher doses were not associated with hypertriglyceridemia. Spline regression modeling for **(B)** LPCH Stanford and **(C)** BIDC to evaluate continuous triglyceride level, adjusted risk of respiratory distress syndrome, bronchopulmonary dysplasia, necrotizing enterocolitis, and retinopathy of prematurity. The model was adjusted for gestational age, birth weight and sex covariates.

In multivariate regression to predict hypertriglyceridemia, GA, preterm labor, and severe IVH were significantly associated with TG values >200 mg/dL and >250 mg/dL (Figure 4A, Supplementary Table S4). Under both definitions of hypertriglyceridemia (200 mg/dL and 250 mg/dL), each additional week of GA was associated with a 30% lower odds of developing hypertriglyceridemia, with 95% CI 0.6–0.8 for levels >200 mg/dL and 0.6–0.9 for levels >250 mg/dL. Preterm labor was associated with a 50% lower odds of hypertriglyceridemia (95% CI 0.3–0.9 for >200 mg/dL, 0.2–1.0 for >250 mg/dL). IVH was associated with an increased risk for both categories including aOR 3.5 (95% CI 1.6–7.9) and 5.2 (95% CI 2.0–13.3), respectively (Figure 4A**)**. There was no observable statistically significant relationship with BW and hypertriglyceridemia risk although, the point estimates demonstrated a slight inverse relationship at 0.9 (95% CI 0.4–1.9) for >200 mg/dL and 0.5 (95% CI 0.1–1.8) for >250 mg/dL. Lower lipid emulsion dose (1 g/kg/d—<2 g/kg/d) was associated with decreased risk for hypertriglyceridemia at thresholds of 200 mg/dL and 250 mg/dL; however, administration of a higher lipid dose did not demonstrate a consistent increase in risk. Postnatal day was not a significant factor in the model. Precision of the model was higher when hypertriglyceridemia was defined as serum TG levels >200 mg/dL (compared to 250 mg/dL), as indicated by lower standard errors and narrower confidence intervals, attributable to a greater number of qualifying events.

Spline regression was used to model the relationship between continuous TG values and infant outcomes with adjustments for GA, BW and sex in both cohorts. There was no generally statistically significant relationship observed for each outcome (Figures 4B,C). Confidence band widths generally increased as TG levels approached 200 mg/dL and beyond.

## Discussion

Our multi-centered investigation of preterm infants <32 weeks' gestation is the largest study to date examining TG levels throughout the postnatal period. We provide insight into several questions that smaller studies have been unable to fully address. These include the following: 1) TG levels and reference ranges were largely consistent with prior investigations, 2) TG levels demonstrated a strong inverse correlation with both GA and BW, but with the highest values observed among neonates born at <25 weeks' gestation and/or within the extremely low birth weight population, 3) higher TG values for <25 week infants remained consistent until 2–3 weeks postnatal age and subsequently reached a nadir at approximately 10 weeks of age, 4) hypertriglyceridemia was a rare and transient event, with multivariable modeling demonstrating decreased risk with the use of lower lipid emulsion dosing and, 5) there was no statistically significant relationship between continuous TG levels and the outcomes of RDS, BPD, NEC, and ROP across either the LPCH Stanford or BIDMC cohorts.

### TG reference ranges for <32 week infants corroborate prior investigations

There are no established TG reference ranges for preterm neonates. Prior investigations in preterm infants have focused on defining hypertriglyceridemia in the context of associations with neonatal outcomes without defining reference ranges. Our results demonstrate age- and weight-specific reference intervals that corroborate prior investigations (22–24). Indeed, such ranges will need to be further validated in larger population-based studies. However, our results provide a data-driven approach for defining reference intervals, especially in the smallest and most vulnerable infants.

### TG values are largely influenced by age-and weight-specific levels and trajectories

Our findings also reinforce established associations between GA, BW and elevated TG values (22, 23, 27–30). However, covariates such as GA and BW have often been aggregated in wide intervals (23, 31), obscuring stark differences in TG levels between older and younger preterm infants. Here, we observe that GA and BW binning in narrow intervals (e.g., 1–2 weeks or 250 g), provides potential insight into developmentally regulated processes such as lipoprotein lipase activity and/or function. Although LOESS modeling demonstrated an inverse association between birth weight and triglyceride concentrations in the LPCH cohort, and was directionally concordant in the multivariate modeling, the association was not statistically significant. Such a result may reflect several factors. First, birth weight is closely correlated with gestational age and neonatal illness severity, and its apparent association with TG levels may be partly confounded by these related variables. Second, the LOESS analysis examined TG as a continuous outcome, whereas the multivariable models evaluated dichotomous predefined hypertriglyceridemia thresholds, which may reduce sensitivity to detect graded differences across the full TG distribution. Third, lipid emulsion dose and postnatal age may mediate or modify the relationship between birth weight and TG tolerance. These findings suggest that lower birth weight is associated with higher TG concentrations descriptively, but that birth weight alone may not independently predict clinically defined hypertriglyceridemia after accounting for other relevant clinical factors. Accordingly, BW-specific normative ranges should be interpreted cautiously and require prospective multi-centered validation.

### Periviable infants have lower TG clearance across postnatal age

Figure 2E demonstrates longitudinal TG trends for infants <25 weeks across postnatal life. Contrary to older infants, we observed that infants in this age category exhibit unique TG metabolism with higher values over the first 2 weeks of life that slowly downtrend. These results are similar to Giretti et al., whereby aggregated mean plasma TG (for the duration of NICU hospitalization) were consistently highest for infants <25 weeks (22). Longitudinal profiling in this population suggests that TG clearance and metabolism, likely mediated by lipoprotein lipase activity in the capillary endothelium (8), is developmentally immature compared to older infants, in the first 2–3 weeks of postnatal life. These results are also supported by broader translational and systems biology investigations that have demonstrated metabolic maturity is highly influenced within specific development windows (20, 32–36).

### Hypertriglyceridemia events are rare and transient

We observed that hypertriglyceridemia events (defined as TG>200 mg/dL and 250 mg/Dl) were rare (6.6% and 3.4% of all values, respectively) and transient (mean 1.73 days). In order to better identify factors associated with these events, we performed logistic regression modeling for hypertriglyceridemia (analysis only for LPCH Stanford). We observed that intravenous lipid dosing at higher ranges was not associated with hypertriglyceridemia. Lower doses (<2 g/kg/d) were associated with a decreased risk for hypertriglyceridemia. These findings suggest that individual lipid emulsion dosing may have only a slight contribution to elevated TG values. Although we did not investigate the timing of Intralipid® dosing advances or changes, we did observe that postnatal day (as a continuous predictor variable) did not significantly impact the regression model. Given the anticipated increase in lipoprotein lipase activity with advancing age, we did not expect this finding. Indeed, lipoprotein lipase activity increases substantially during the first 24 h after birth and continues to develop to adult levels over time, varying by tissue type (37, 38). From a clinical perspective, the data in aggregate confirm efficacy for the use of commonly known clinical strategies to reduce TG levels, namely, maintaining vigilance over intravenous dosing. Note that the precision of our estimates was greater when hypertriglyceridemia was defined using a threshold of >200 mg/dL, likely due to a higher number of qualifying events, resulting in narrower confidence intervals and more stable model estimates.

Severe hypertriglyceridemia can cause pseudohyponatremia, in which high triglyceride levels interfere with sodium measurement, leading to falsely low sodium values (25, 26). Pseudohyponatremia may not require intervention. However, if clinicians misinterpret the falsely low sodium as true hyponatremia and attempt correction with sodium administration or fluid restriction, these interventions may cause harm, potentially resulting in true hypernatremia or dehydration. Monitoring triglyceride levels may thus assist in identtification of pseudohyponatremia and the prevention of unnecessary sodium repletion and/or fluid restriction.

### TG values have No discernable relationship with RDS, BPD, NEC and ROP

We observed no consistent relationship in either the LPCH Stanford or the BIMDC cohort between TG values and the infant outcomes of RDS, BPD, NEC or ROP. In hypertriglyceridemia regression modeling exclusively at LPCH Stanford, there was a relationship between hypertriglyceridemia and severe IVH. This finding is corroborated by prior investigations that have demonstrated associations between elevated TG values and co-occurring critical illness (23, 30). However, beyond IVH, we argue that relationships between TG levels and outcomes remain difficult to fully explore given the relative lack of hypertriglyceridemia events, the relatively low numbers of patients with specific outcomes (e.g., NEC) and the relative paucity of TG levels consistently in specific ranges, (i.e., <40 mg/dL and/or >200 mg/dL). However, our results suggest that across continuous TG levels, there was no consistent relationship with interrogated outcomes. The lack of an association suggests that impaired TG and/or lipid metabolism may not play a central role in later infant outcomes. However, prior work suggests that lipid tolerance is negatively associated with major complications of prematurity, including severe intraventricular hemorrhage, and late-onset sepsis (7, 22, 39, 40). In this context, an elevated TG level compared to baseline, may serve as a marker of impaired TG clearance due to systemic illness. Indeed, there is evidence that intraindividual changes in laboratory levels from an established baseline may be associated with elevated risk for common diseases including myocardial infarction, stroke, diabetes, kidney disease and osteoporosis (41).Furthermore, the long-term cardiovascular and metabolic effects of early-life lipid levels remain incompletely understood. A study of young adults born prematurely found that those who received intravenous lipids as neonates had greater aortic stiffness and reduced myocardial function compared to unexposed neonates (42). Preterm birth itself is associated with increased risk of lipid disorders in early adulthood (43), and former very preterm infants have unfavorable cardiovascular risk profiles in early childhood and adulthood, including hypercholesterolemia and systolic prehypertension as early as five years old (43, 44). Thus, routine TG monitoring enables the establishment of a laboratory baseline that when altered, may warrant additional interventions.

Overall, this study highlights the dynamic nature of TG metabolism among preterm infants during the early postnatal period, influenced by gestational age and birth weight. The findings suggest that while lower GA is a significant risk factor for higher TG values, cautious advancement of lipid dosing does not inherently increase risk for hypertriglyceridemia or for associated infant outcomes. These results reinforce the importance of individualized lipid management strategies in the NICU setting, balancing the need for adequate dosing with careful monitoring. Further prospective studies are warranted to define optimal TG thresholds that support growth and development while minimizing metabolic complications in this vulnerable population.

### Limitations

There were several limitations in this study. First, this retrospective observational study was conducted in only two academic center cohorts and therefore our findings may not be broadly generalizable. The data extraction methods between the two cohorts was distinct, with slight differences in the curation and subsequent analysis of such data. In addition, there was limited TG sampling beyond the first two weeks postnatal age in the LPCH Stanford cohort. Further, the number of hypertriglyceridemia events across both cohorts was limited making it difficult to interpret findings with respect to this outcome. Within the BIDMC cohort, the postnatal timing of TG sampling was not available. In addition, covariates such as GA and BW were unavailable and/or missing from some infants in this cohort, requiring the use of imputation techniques in a subset of infants.

## Conclusion

Our findings highlight the relatively rare and transient nature of hypertriglyceridemia across infants <32 weeks in aggregate. Across all TG values, only 6.6% were >200 mg/dL and 3.4% were >250 mg/dL. However, we observed prolonged metabolic immaturity of infants born before 25 weeks' gestation, a group that demonstrates the highest susceptibility to lipid metabolism perturbations. These infants likely represent a metabolically distinct subgroup, as borne out by prior literature (22, 45). Serum TG levels were strongly associated with GA and weight, rather than being primarily driven by intravenous lipid administration. Beyond severe IVH there were no significant risks for hypertriglyceridemia and infant outcomes, albeit with limitations secondary to the limited number of hypertriglyceridemia events. These insights underscore the need for further mechanistic and longitudinal studies to refine our understanding of early lipid metabolism and to guide personalized approaches to neonatal nutrition and care.

## Data Availability

The original contributions presented in the study are included in the article/Supplementary Material, further inquiries can be directed to the corresponding author/s.
