# Supplementary material for: Triglyceride profiles in preterm infants reveal dynamic and age-specific trajectories
Source: Front Pediatr. 2026 Jun 12;14:1777798. doi: 10.3389/fped.2026.1777798 (PMC13303854; doi:10.3389/fped.2026.1777798)

**Supplemental Table 1**

**Supplemental Table 2**

**Table 3. ICD Codes Used for BIDMC Cohort**

| **Code** | **Version** | **Clinical Diagnosis** |
| --- | --- | --- |
| **76511** | 9 | Other preterm infants, less than 500 grams |
| **76512** | 9 | Other preterm infants, 500-749 grams |
| **76513** | 9 | Other preterm infants, 750-999 grams |
| 76514 | 9 | Other preterm infants, 1,000-1,249 grams |
| **76515** | 9 | Other preterm infants, 1,250-1,499 grams |

| **76501** | 9 | Extreme immaturity, less than 500 grams |
| --- | --- | --- |
| **76502** | 9 | Extreme immaturity, 500-749 grams |
| **76503** | 9 | Extreme immaturity, 750-999 grams |
| **76504** | 9 | Extreme immaturity, 1,000-1,249 grams |
| **76505** | 9 | Extreme immaturity, 1,250-1,499 grams |

| **P0721** | 10 | Extreme immaturity of newborn, gestational age less than 23 completed weeks |
| --- | --- | --- |
| **P0722** | 10 | Extreme immaturity of newborn, gestational age 23 completed weeks |
| **P0723** | 10 | Extreme immaturity of newborn, gestational age 24 completed weeks |
| **P0724** | 10 | Extreme immaturity of newborn, gestational age 25 completed weeks |
| **P0725** | 10 | Extreme immaturity of newborn, gestational age 26 completed weeks |
| **P0726** | 10 | Extreme immaturity of newborn, gestational age 27 completed weeks |
| **P0731** | 10 | Preterm newborn, gestational age 28 completed weeks |
| **P0732** | 10 | Preterm newborn, gestational age 29 completed weeks |
| **P0733** | 10 | Preterm newborn, gestational age 30 completed weeks |
| **P0734** | 10 | Preterm newborn, gestational age 31 completed weeks |

| **Code** | **Version** | **Disease** |
| --- | --- | --- |
| **7707** | 9 | BPD |
| **P271** | 10 | BPD |
| **77750** | 9 | NEC |
| **77751** | 9 | NEC |
| **77752** | 9 | NEC |
| **77753** | 9 | NEC |
| **P770** | 10 | NEC |
| **P771** | 10 | NEC |
| **P772** | 10 | NEC |
| **P773** | 10 | NEC |
| **P779** | 10 | NEC |
| **36220** | 9 | ROP |
| **36221** | 9 | ROP |
| **36222** | 9 | ROP |
| **36223** | 9 | ROP |
| **36224** | 9 | ROP |
| **36225** | 9 | ROP |
| **36226** | 9 | ROP |
| **36227** | 9 | ROP |
| **H3510** | 10 | ROP |
| **H3511** | 10 | ROP |
| **H3512** | 10 | ROP |
| **H3513** | 10 | ROP |
| **H3514** | 10 | ROP |
| **H3515** | 10 | ROP |
| **H3516** | 10 | ROP |

**Supplemental Table 4**


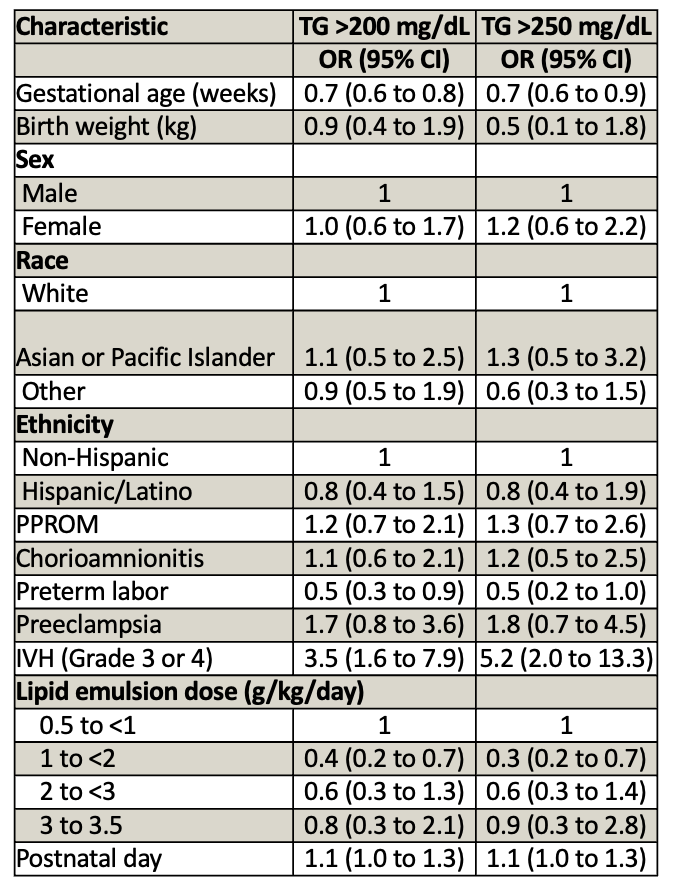

Supplement: Supplementary file 1 [file Supplementaryfile1.docx]
